# Le carcinome verruqueux du lit ungueal: entité rare du carcinome épidermoide

**DOI:** 10.11604/pamj.2013.16.135.3558

**Published:** 2013-12-11

**Authors:** Noama Dahbi, Said Amal

**Affiliations:** 1 Service de Dermatologie, CHU Mohammed VI, Université de Médecine et de Pharmacie, Marrakech, Maroc

**Keywords:** Carcinome verruqueux, lit ungueal, carcinome épidermoide, verrucous carcinoma, ungueal bed, epidermoid carcinoma

## Images in medicine

Le carcinome verruqueux du lit unguéal est rare et le retard diagnostique est fréquent d'où l'intérêt de biopsier les lésions unguéales chroniques. Sa localisation fréquente est au niveau des muqueuses, des doigts surtout le pouce, rarement les orteils et plus rarement le siège sous unguéale. Il survient surtout chez le sujet âgé de plus de 60 ans avec prédominance masculine. Plusieurs facteurs sont incriminés dans sa pathogénie: l'exposition aux radiations ionisantes, les radiodermites chroniques, les microtraumatismes répétés, et l'infection chronique à HPV. Il est de bon pronostic, à malignité locale. Les métastases viscérales sont exceptionnelles. L'approche thérapeutique n'est pas standardisée à l'heure actuelle. Le traitement conservateur est préférentiel afin de préserver les fonctions de l'orteil. L'amputation de la phalange distale est réalisée généralement en cas d'atteinte osseuse. Les récidives sont fréquentes d'où l'intérêt d'une exérèse large et un suivi prolongé des patients. Nous rapportons l'observation d'une patiente âgée de 60 ans, sans antécédents pathologiques particuliers, qui présentait depuis 4 ans, une tumeur verruqueuse sous unguéale du gros orteil gauche, sans adénopathies palpables, faisant évoquer une verrue vulgaire, une tuberculose verruqueuse, une maladie de Bowen ou une mycose profonde. La radiologie standard de l'orteil était normale. La biopsie cutanée était en faveur d'un carcinome verruqueux. La patiente a bénéficié d'une exérèse du lit de l'ongle et de la matrice avec limites d'exérèses saines à l’étude histologique. Une greffe de peau totale est réalisée après bourgeonnement. Le recul est de 2 ans.

**Figure 1:**
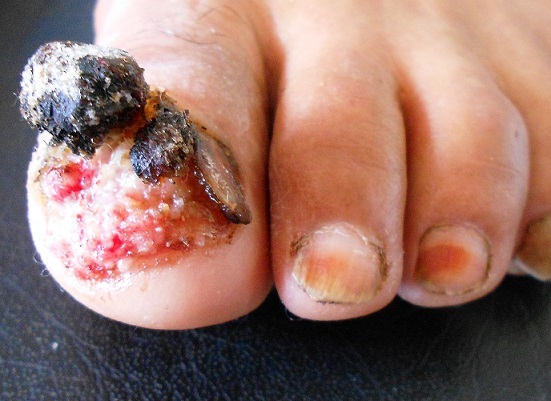
Lésion verruqueuse sous unguéale

